# Health Tract, No. 19

**Published:** 1865

**Authors:** 


### HEALTH TRACT, No. 19.

### WEARING FLANNEL.

In our climate, fickle in its gleams of sunshine and its balmy airs, as a coquette with her smiles and favors, consumption bears away every year the ornaments of many social circles. The fairest and loveliest are its favorites. An ounce of prevention in this fatal disease is worth many pounds of cure, for when once well seated, it mocks alike medical skill and careful nursing. If the fair sex could be induced to regard the laws of health, many precious lives might be saved; but pasteboard soles, the low-neck dresses, and lilliputian hats, sow annually the seeds of a fatal harvest. The suggestion in the following article from the Journal of Health, if followed, might save many with consumptive tendencies from an early grave:

"Put it on at once; winter and summer nothing better can be worn next to the skin than a loose red woollen shirt; 'loose,' for it has room to move on the skin, thus causing a titillation which draws the blood to the surface and keeps it there; and when that is the case no one can take cold; 'red,' for white flannel fulls up, mats together, and becomes tight, stiff, heavy and impervious. Cotton-wool merely absorbs the moisture from the surface, while woollen flannel conveys it from the skin and deposits it in drops on the outside of the shirt, from which the ordinary cotton shirt absorbs it, and by its nearer exposure to the air it is soon dried without injury to the body. Having these properties, red wool flannel is worn by sailors even in the midsummer of the warmest countries. Wear a thinner material in summer."

### TO CONSUMPTIVES.

You want air, not physic; you want pure air, not medicated air; you want nutrition, such as plenty of meat and bread will give, and they alone; physic has no nutriment; gasping for air can not cure you; monkey-capers in a gymnasium can not cure you; and stimulants can not cure you. If you want to get well, go in for *beef and out-door air*, and do not be deluded into the grave by advertisements and unreliable certifiers.

### THREE ESSENTIALS.

The three great essentials to human health are: Keep the feet always dry and warm; Have one regular action of the bowels every day; and Cool off very slowly after all forms of exercise.

